# Dataset for stacking-mediated diffusion of ruthenium nanoclusters in bilayer graphene and graphite

**DOI:** 10.1016/j.dib.2022.108702

**Published:** 2022-10-26

**Authors:** James G. McHugh, Pavlos Mouratidis, Kenny Jolley

**Affiliations:** a Dept. of Physics and Astronomy, University of Manchester, Oxford Road, Manchester M13 9PL, United Kingdom; b National Graphene Institute, University of Manchester, Booth St E, Manchester M13 9PL, United Kingdom; c Dept. of Chemistry, Loughborough University, Epinal Way, Loughborough LE11 3TU, United Kingdom

**Keywords:** Density functional theory, Graphite, Graphene, Bilayer graphene, Intercalation, Surface diffusion, Fission products, Transition metals

## Abstract

The data in this article are related to the research article “Stacking-Mediated Diffusion of Ruthenium Nanoclusters in Bilayer Graphene and Graphite” (J G McHugh, 2022). The data consists of Ru atom cluster intercalation calculations on graphene surfaces, within AA/AB bilayer graphene and graphite. We tabulate data for cluster sizes of 3, 4, 5 and 7 Ru atoms, which includes adsorption energies and diffusion energy barriers between all the highly symmetric sites in graphene/graphite. These data were obtained from density functional theory calculations. We provide tabulated data of relaxed structures that are useful for future classical interatomic potential fittings.

**Specifications Table**

**Table:** 

Subject	Computational materials science
Specific subject area	Structure of Ru clusters in graphite for nuclear and device-engineering applications.
Type of data	Images, Tables, Structural data.
How the data were acquired	The data was acquired by using high performance computing facilities (HPC). Density functional theory (DFT) calculations were performed using the Quantum Espresso code (https://www.quantum-espresso.org). The input files were generated by placing clusters of ruthenium atoms placed at a selection of high symmetry positions. Setting up all the required parameters in the DFT code, through a geometry optimization it finds the minima energy configuration for each structure. Finally, we have extracted in xyz format the optimized geometry configurations.
Data format	Analyzed structures given as rendered images. Analyzed numerical data of the adsorption energies and transition energy barriers are tabulated. Raw atomic coordinates of the relaxed structures in xyz format are available on the Mendeley data repository.
Description of data collection	Output from quantum espresso calculations of structural, energetic and nudged elastic band diffusion barriers for ruthenium on graphene and in graphite for a variety of different initial conditions.
Data source location	Department of Chemistry, School of Science, Loughborough University, Loughborough, LE11 3TU, United Kingdom (Latitude: 52.762000°, Longitude: -1.241000°)
Data accessibility	Repository name: Mendeley Data Data identification number: DOI: 10.17632/78jc73v6vg.1 Direct URL to data: https://data.mendeley.com/datasets/78jc73v6vg/1
Related research article	James G. McHugh, Pavlos Mouratidis, Kenny Jolley, Stacking-mediated diffusion of ruthenium nanoclusters in bilayer graphene and graphite, Applied Surface Science, Volume 607,2023,154912, ISSN 0169-4332, https://doi.org/10.1016/j.apsusc.2022.154912

## Value of the Data

• The data includes fully relaxed atomic structures and transition coordinates of dynamical processes from fully quantum mechanical simulations.
• Pre-relaxed positions are useful as initial positions and conditions in simulations.
• This data is useful for computational researchers performing other simulations or structural search procedures of transition metals on graphene.
• These data could also be used as part of future interatomic potential fittings by generating initial structures.
• Experimentalists can use this type of structural data to aid interpretation of relevant experiments.

## 1 Data Description

The raw structural data, consisting of initial conditions, final conditions, and transition state coordinates from fully quantum mechanical DFT calculations of ruthenium adatoms on graphene monoloayer (ML), graphene bilayer (BL) and graphite have been uploaded to the associated data repository.

**Fig. 1:**
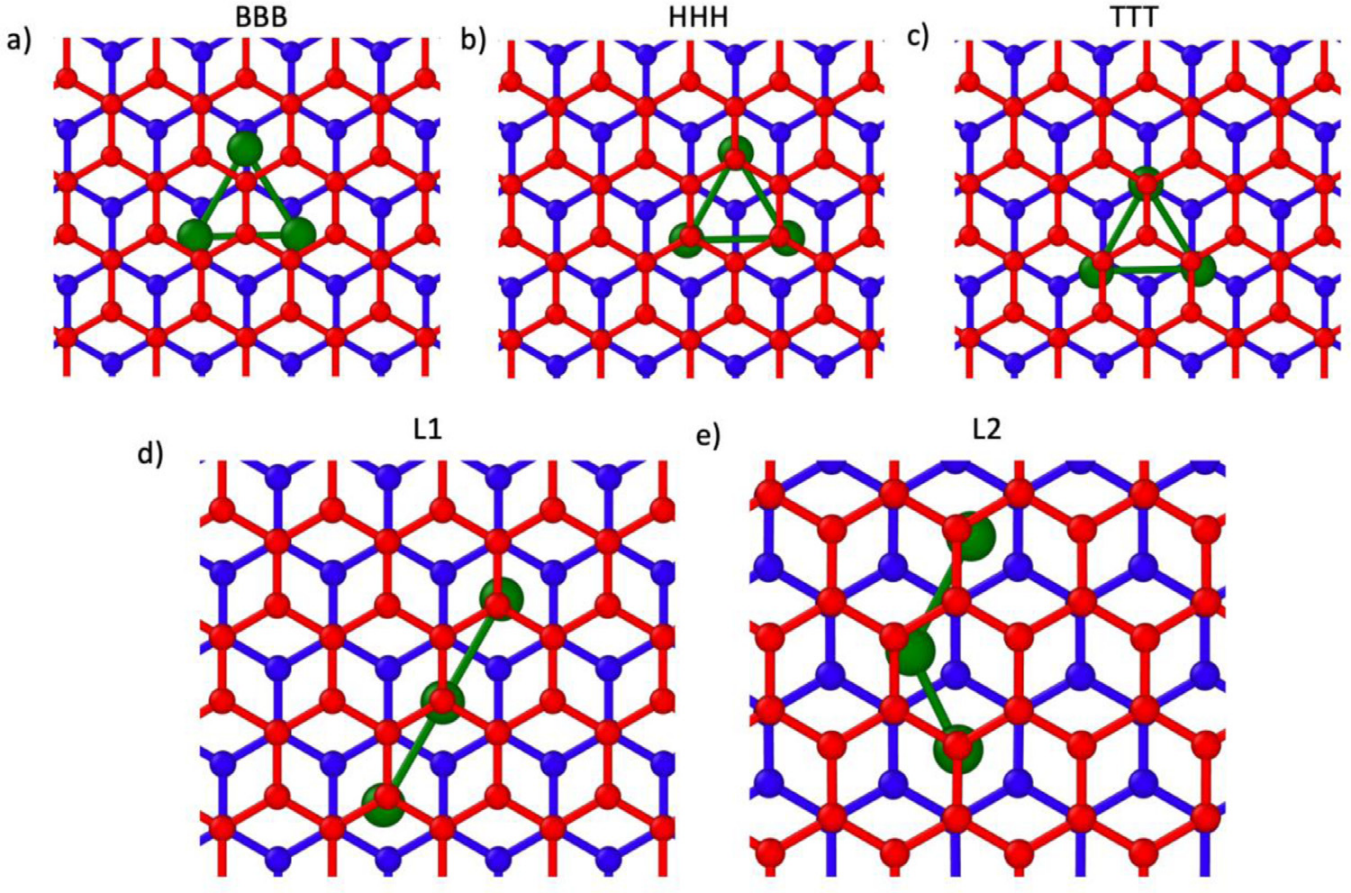
Initial positions of Ru n = 3 nanocluster simulations in an AB-stacked bilayer, with the A and B oriented layers colored blue and red respectively, and the Ru atoms colored green.

**Fig. 2:**
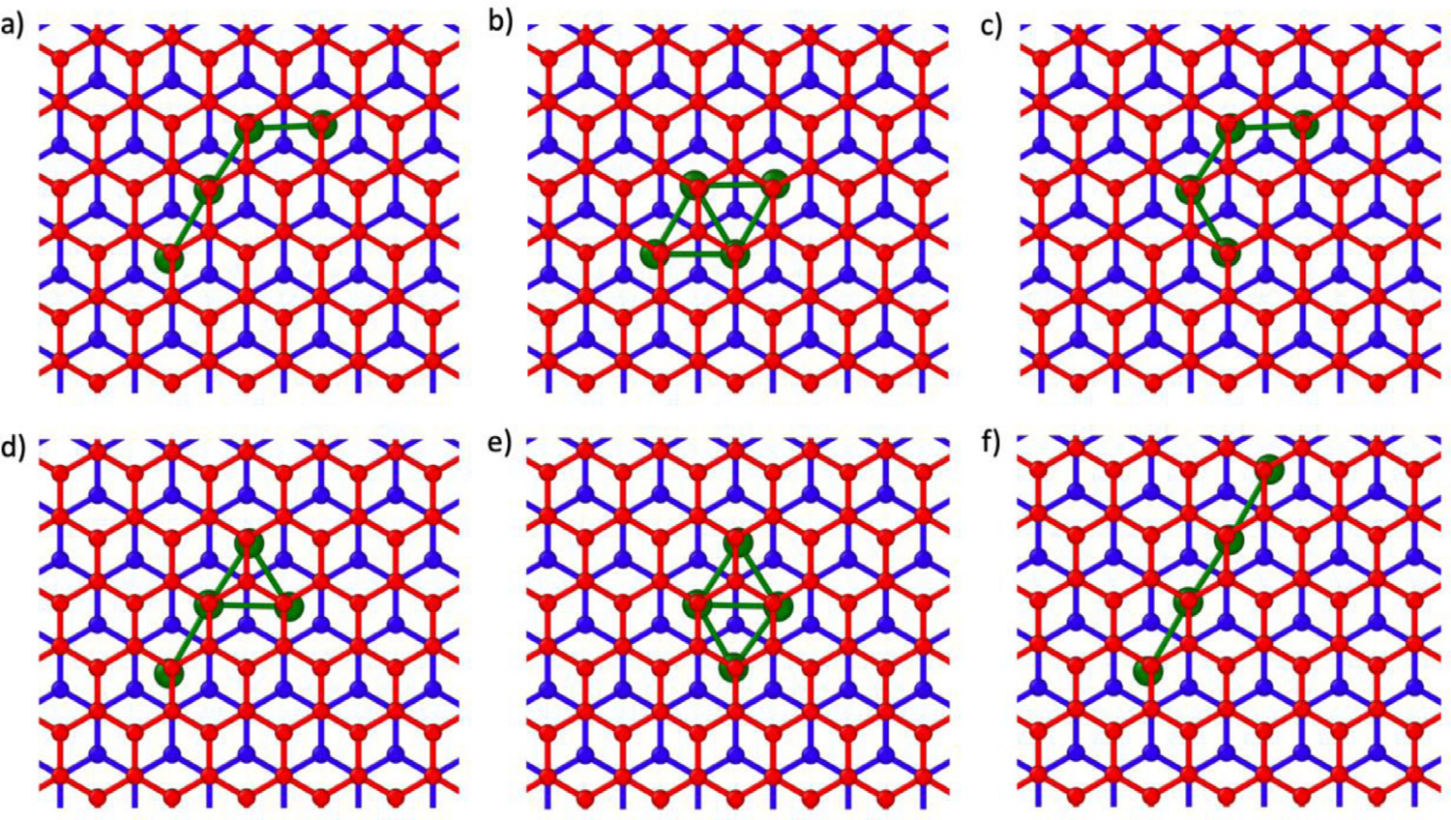
Initial positions of n = 4 nanoclusters in AB BL graphene. The A and B oriented layers are colored blue and red respectively and the Ru atoms are colored green.

**Fig. 3:**
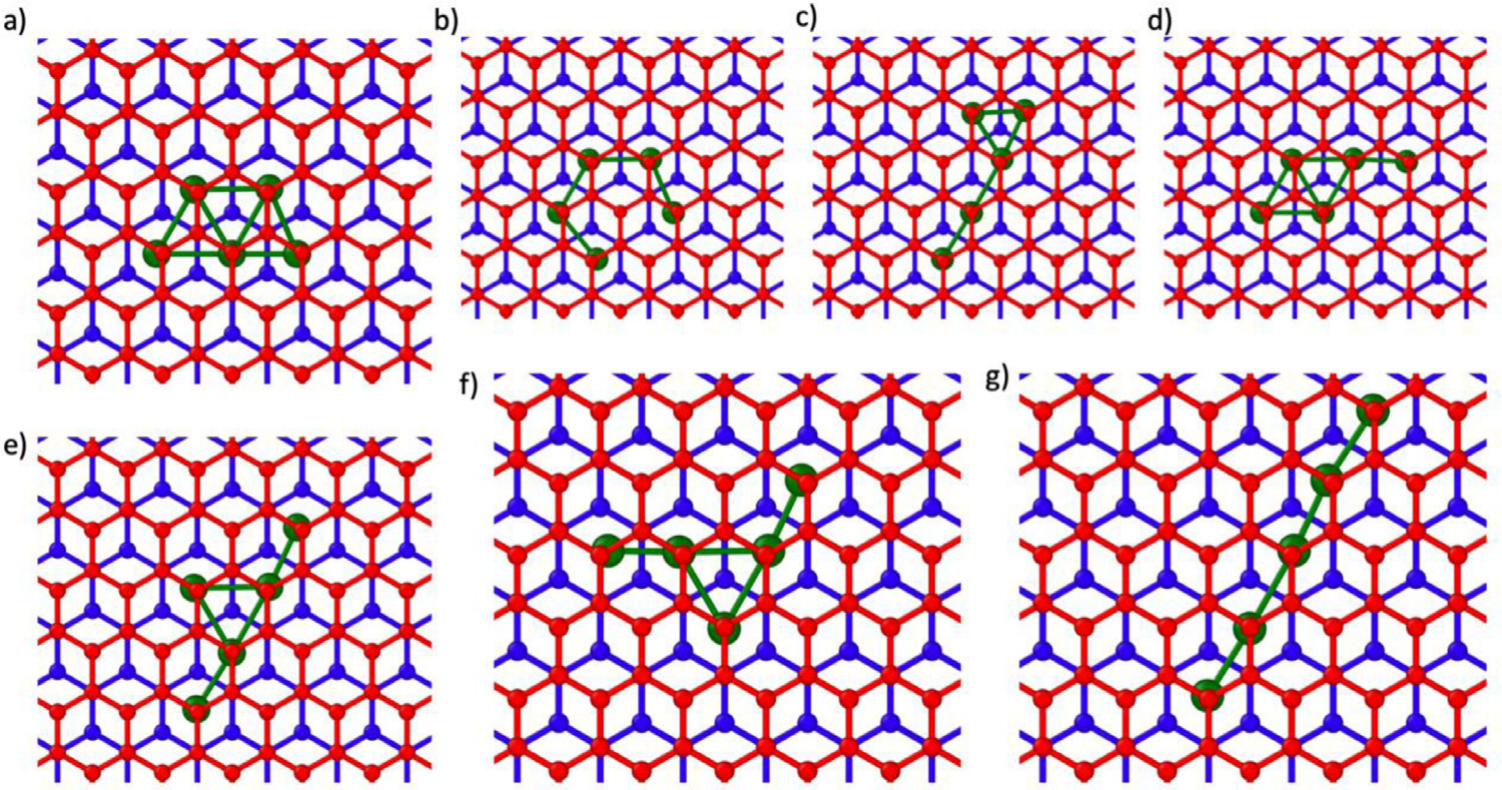
Initial positions of n = 5 nanoclusters in AB BL graphene. The A and B oriented layers are colored blue and red respectively and the Ru atoms are colored green.

**Fig. 4:**
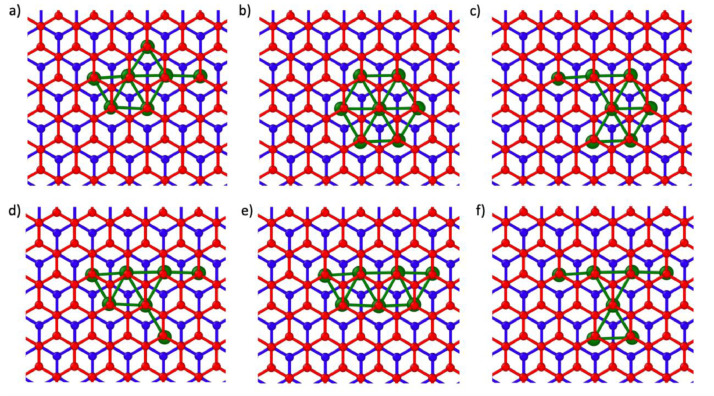
Initial positions of n = 7 nanoclusters in AB BL graphene, The A and B oriented layers are colored blue and red respectively and the Ru atoms are colored green.

Initial positions of Ru clusters of size 3, 4, 5, and 7 are depicted in the rendered images shown in Figs. 1-4 respectively.

**Fig. 5:**
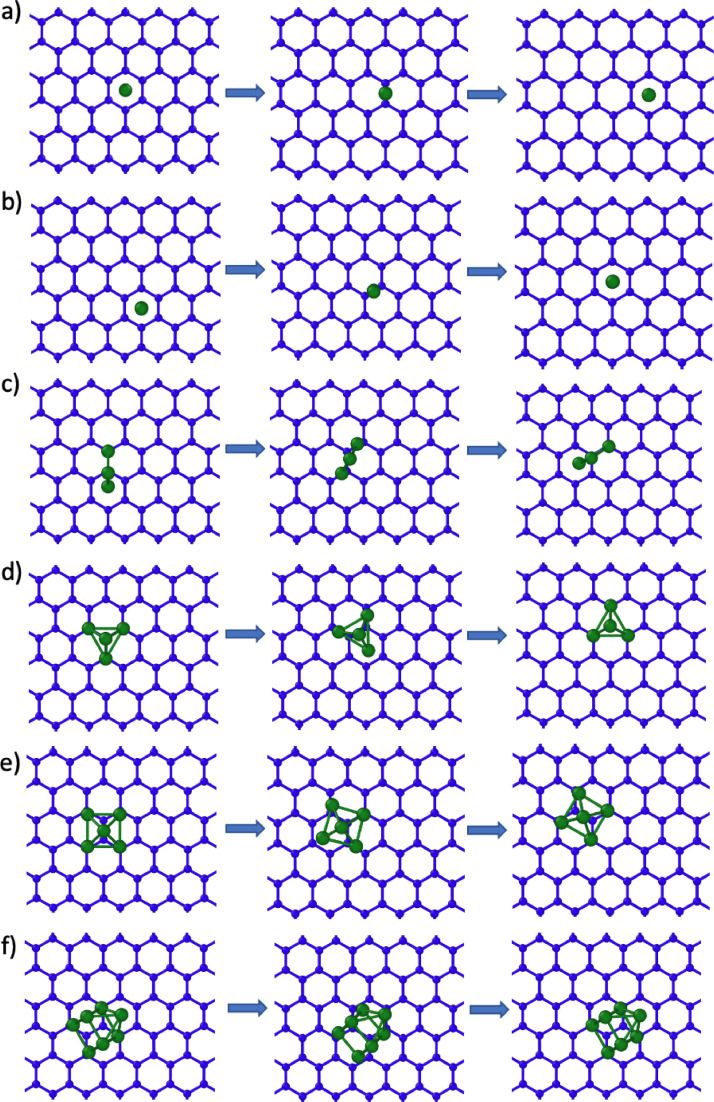
Transition barrier pathways for n = 1, 2, 3, 4, 5, 7 Ru nanocluster diffusion on monolayer graphene. The initial, saddle point and final state is shown. The graphene monolayer is colored blue and the Ru atoms are colored green.

**Fig. 6:**
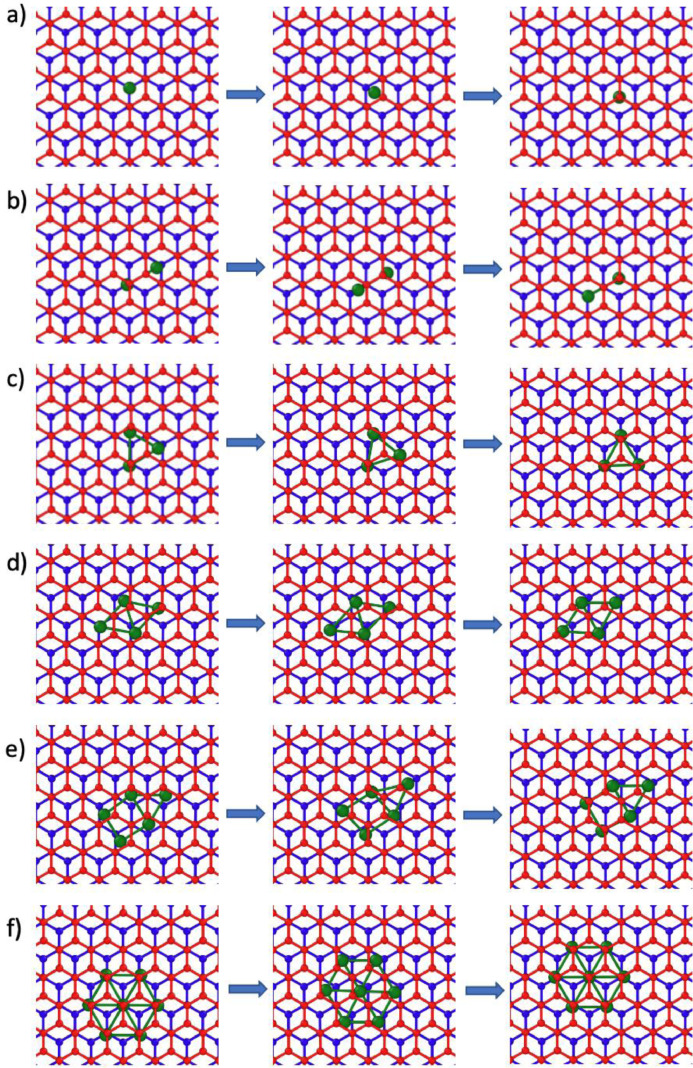
Transition pathways for n = 1, 2, 3, 4, 5, 7 Ru nanocluster diffusion on AB BL graphene. The initial, saddle point and final state is shown. The A and B oriented layers are colored blue and red respectively and the Ru atoms are colored green.

**Fig. 7:**
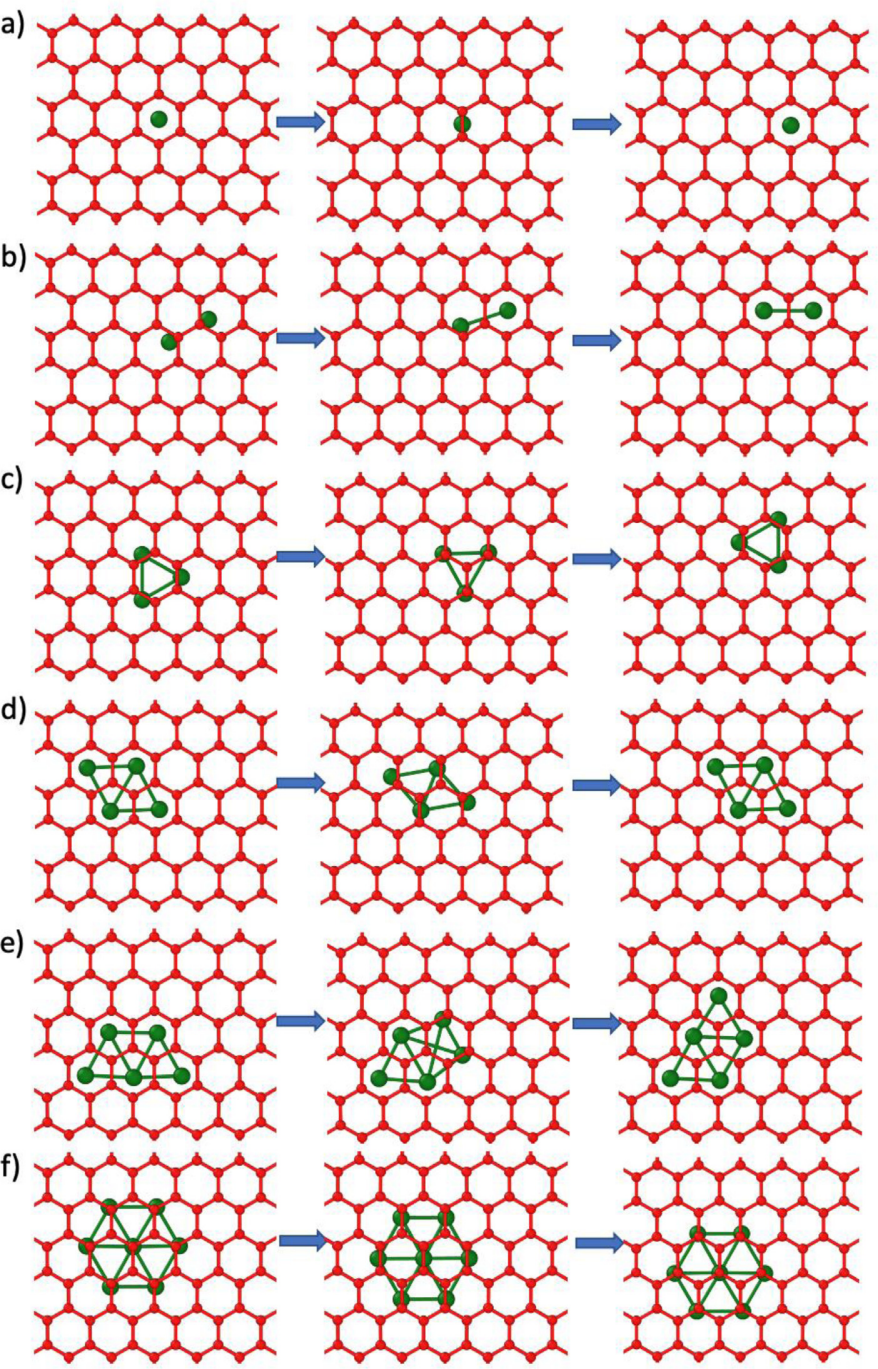
Transition pathways for n = 1, 2, 3, 4, 5, 7 Ru nanocluster diffusion on AA BL graphene. The initial, saddle point and final state is shown. The A and B oriented layers are colored blue and red respectively and the Ru atoms are colored green.

Figs. 5–7 show rendered images of the initial, saddle point and final states of the transition pathways for the Ru clusters. These are given for the ML graphene and AB/AA BL graphene.

For Figs. 1-4, the corresponding raw xyz coordinates can be found within the Mendeley data repository. The folder structure is sorted by the Ru atom cluster size (ie folder 1 contains all structures with a single Ru atom). Within these folders, subfolders contain structures for the monolayer (ML), bilayer (BL) and bulk graphite (Bulk). Both AA and AB stacking configurations are available. The Mendeley data also contains raw xyz coordinates for the full nudged elastic band (NEB) pathways shown in Figs. 5–7.

Further data includes values of adsorption energies and transition state energy barriers for all investigated structures, which are documented in Table 1, Table 2, Table 3, Table 4, Table 5, Table 6, Table 7, Table 8, Table 9, Table 10, Table 11, Table 12, Table 13, showing the adsorption and intercalation energies of Ru nanocluster on graphene and graphite. Table 1-6 shows adsorption energies of Ru nanoclusters of sizes n=1,2,3,4,5,7 respectively, for the different initial positions.

**Table 1:** Adsorption energies of Ru adatoms on graphene for different initial positions.

Site	E_Ads_ (eV)	Mag (μB)	Height (A)	∆E
T	5.49	1.69	1.91	0.67
B	5.52	1.77	2.00	0.70
H	4.82	1.77	1.68	0.00

**Table 2:** Adsorption energies of Ru dimers on graphene for different initial positions.

Site	E_Ads_ (eV)	Mag (μB)	∆E
TT1	4.922	0.000	0.863
TT2	4.452	1.775	0.393
TT3	4.640	0.000	0.581
BB1	4.059	2.000	0.000
BB2	4.461	0.000	0.402
BB3	4.672	0.000	0.613
HB	4.601	1.955	0.542
HH	4.528	0.000	0.469

**Table 3:** Adsorption energies of n=3 Ru clusters on graphene.

Site	E_Ads_ (eV)	Mag (μB)	∆E
TTT	3.737	1.99	0.272
BBB	3.759	0.57	0.294
HHH	3.571	0.67	0.106
L1	3.688	0.69	0.223
L2	3.465	0.62	0.000

**Table 4:** Adsorption energies of n=4 Ru clusters on graphene.

Site	E_Ads_ (eV)	Mag (μB)	∆E
A	3.193	0.975	0.000
B	3.679	0.540	0.486
C	3.346	1.020	0.153
D	3.337	1.378	0.144
E	3.250	1.025	0.057

**Table 5:** Adsorption energies of n=5 Ru clusters on graphene.

Site	E_Ads_ (eV)	Mag (μB)	∆E
A	2.92	0.468	0.091
B	2.829	0.010	0.000
C	2.907	0.642	0.078
D	2.920	0.468	0.091
E	3.211	1.054	0.382
F	2.920	0.140	0.091
G	3.420	0.000	0.591

**Table 6:** Adsorption energies of n=7 Ru clusters on graphene.

Site	E_Ads_ (eV)	Mag (μB)	∆E
A	2.498	1.044	0.237
B	2.527	0.444	0.266
C	2.261	0.283	0.000
D	2.381	0.000	0.120
E	2.559	0.444	0.298
F	2.622	0.562	0.361

Table 7 shows intercalation energies of a single Ru atom in AA & AB stacked BL graphene and bulk graphite. Tables 8, 9 show intercalation energy for n=2 clusters in AA & AB stacked BL graphene and bulk graphite, respectively. Table 10, Table 11, Table 12, Table 13 shows intercalation energies of n=3, 4, 5, 7 clusters in AA & AB BL graphene.

**Table 7:** Single Ru atom intercalation, bilayer and bulk adsorption energies.

	Bilayer			Bulk		
Site	E_Ads_ (eV)	Mag (μB)	∆E	E_Ads_ (eV)	Mag (μB)	∆E
T (AA)	1.68	1.85	0.0182	1.8702	0.000	0
B (AA)	1.6618	0.000	0.000	1.9499	0.000	0.0797
H (AA)	3.6224	0.000	1.9606	3.8209	0.000	1.9507
T (AB) (T1,T2)	2.3614	1.610	0.6996	2.5251	0.000	0.6549
B (AB)	2.405	1.620	0.7432	3.2203	0.000	1.3501
H (AB) (T1,H2)	3.2022	0.000	1.5404	2.28	0.000	0.4098

**Table 8:** Ru dimer, AA bilayer and bulk intercalation energies.

	Bilayer		Bulk	
Site	E_Ads_ (eV)	∆E	E_Ads_ (eV)	∆E
TT1	3.584	0.208	2.993	0.004
TT2	3.809	0.433	2.993	0.004
TT3	3.584	0.208	2.993	0.004
BB1	3.844	0.468	2.989	0.000
BB2	3.584	0.208	2.989	0.000
BB3	3.811	0.435	2.989	0.000
HH	3.376	0.000	2.989	0.000

**Table 9:** Ru dimer, AB bilayer and bulk intercalation energies.

	Bilayer		Bulk	
Site	E_Ads_ (eV)	∆E	E_Ads_ (eV)	∆E
TT1	3.346	0.112	3.294	0.130
TT2	3.80	0.566	3.522	0.358
TT3	3.348	0.114	3.298	0.134
BB1	3.313	0.079	3.734	0.570
BB2	3.269	0.035	3.207	0.043
BB3	3.268	0.034	3.207	0.043
HH	3.234	0.000	3.164	0.000

**Table 10:** Ru n=3 cluster, AA and AB bilayer intercalation energies.

	AA		AB	
Site	E_Ads_ (eV)	∆E	E_Ads_ (eV)	∆E
TTT	3.326	0.191	3.227	0.473
BBB	3.359	0.224	3.019	0.265
HHH	3.228	0.093	3.227	0.473
L1	3.214	0.079	2.836	0.082
L2	3.135	0.000	2.754	0.000

**Table 11:** Ru n=4 cluster, AA and AB bilayer intercalation energies.

	AA		AB	
Site	E_Ads_ (eV)	∆E	E_Ads_ (eV)	∆E
A	3.053	0.000	2.803	0.000
B	3.393	0.340	3.189	0.386
C	3.328	0.275	3.187	0.384
D	3.178	0.125	3.065	0.262
E	3.231	0.178	2.839	0.036

**Table 12:** Ru n=5 cluster, AA and AB bilayer intercalation energies.

	AA		AB	
Site	E_Ads_ (eV)	∆E	E_Ads_ (eV)	∆E
A	3.055	0.000	2.672	0.006
B	3.062	0.007	2.666	0.000
C	3.109	0.054	2.746	0.080
D	3.221	0.166	2.810	0.144
E	3.215	0.160	3.025	0.359
F	3.079	0.024	2.779	0.113
G	3.395	0.340	3.128	0.462

**Table 13:** Ru n=7 cluster, AA and AB bilayer intercalation energies.

	AA		AB	
Site	E_Ads_ (eV)	∆E	E_Ads_ (eV)	∆E
A	2.921	0.351	2.498	0.117
B	2.574	0.004	2.527	0.146
C	2.574	0.004	2.6	0.219
D	2.57	0.000	2.381	0.000
E	2.597	0.027	2.559	0.178
F	2.643	0.073	2.622	0.241

## 2 Experimental Design, Materials and Methods

The corresponding geometries (xyz files) of different graphene, bilayer-graphite stacking configurations were generated through Python scripts. Then the Ru atoms were placed on the specified high symmetry positions. Next, the geometries were optimized within the DFT method using the Quantum ESPRESSO ab-initio package [1], following the same methodology as in our previous work [2]. The exchange-correlation functional which treats the valence electron-electron interactions was approximated by the generalized gradient approximation (GGA) functional, as parameterized by Perdew, Burke, and Ernzerholf (PBE) [3]. The core electrons and nuclei charges were approximated by the Vanderbilt ultrasoft pseudopotentials [4]. The electron wavefunction was expanded as a series of plane-wave basis sets with a maximum cut-off of E_cut_ = 40 Ry (544 eV), and a charge density cut-off of E_ρ_ = 500 Ry (6803 eV). These cut-off energies set the limit on the number of plane wave functions being utilized as basic functions to represent the wavefunction and the charge density. The values chosen, have been found to be in close agreement to the more highly converged calculations. A non-zero electron temperature of k_B_T = 0.02 eV was applied to aid convergence of the optimization algorithms, alongside a Gaussian smearing function. The Brillouin zone was sampled using a Monkhorst-Pack 5×5×1 k -point grid for all the calculations.

The interlayer van der Waals interactions between the graphene layers has been modeled within the Grimme DFT-D2 method [5]. To avoid any spurious self-interactions between periodically repeated images, a vacuum of 20 Å along the z-direction has been used. Structural optimizations were performed until the residual force on each atom is less than 0.01 eV/Å and the energy difference between subsequent iterations is less than 0.01 eV. The adsorption energy per atom (chemical potential) has been calculated as:

$$\mu \left(n\right)\;=\left(E_{tot\;}-E_{Gr\;}\right)/n\;-E_{Ru,\;FCC},$$

where E_Gr_ is the energy of the respective perfect graphitic material (monolayer and AB stacked bilayer graphene), E_Ru,FCC_ is the energy of bulk FCC Ru lattice, taken from a well-converged bulk calculation sampled with a 21 × 21 × 21 k -point grid, E_tot_ is the total energy of the fully-optimized combined Ru nanocluster/graphite system, and n is the number of adsorbed or intercalated Ru atoms.

To facilitate the comparison of energies between different bilayer stacking configurations (different lateral offsets between the graphene layers), we have calculated all intercalation energies relative to the AB-stacked minimum. This expression gives the relative energy per Ru adatom of different ruthenium-carbon configurations. It is defined such that energetically preferred structures have lower formation energy, and configurations with μ < 0 are preferred over bulk Ru. In this way, our data provides full insight into the relative preference for adsorption, intercalation, and clustering both on the monolayer graphene surface and intercalated into the bilayer or bulk lattice [6].

## Ethics Statements

Our work complies with the relevant Data in Brief guidelines for Authors.

## CRediT authorship contribution statement

**James G. McHugh:** Conceptualization, Methodology, Formal analysis, Visualization, Writing – original draft. **Pavlos Mouratidis:** Formal analysis, Visualization, Writing – original draft. **Kenny Jolley:** Visualization, Supervision, Writing – original draft.

## Declaration of Competing Interest

The authors declare that they have no known competing financial interests or personal relationships that could have appeared to influence the work reported in this paper.

## Data Availability

Dataset for Stacking-Mediated Diffusion of Ruthenium Nanoclusters in Bilayer Graphene and Graphite (Original Data) (Mendeley Data).
